# Exploring the Pivotal Neurophysiologic and Therapeutic Potentials of Vitamin C in Glioma

**DOI:** 10.1155/2021/6141591

**Published:** 2021-12-14

**Authors:** Seidu A. Richard, Marian Sackey, Nii Korley Kortei

**Affiliations:** ^1^Department of Medicine, Princefield University, P. O. Box MA-128, Ho, Ghana; ^2^Department of Pharmacy, Ho Teaching Hospital, P.O. Box MA-374, Ho, Ghana; ^3^Department of Nutrition and Dietetics, School of Allied Health Sciences, University of Health and Allied Sciences, Ho, Ghana

## Abstract

Gliomas represent solely primary brain cancers of glial cell or neuroepithelial origin. Gliomas are still the most lethal human cancers despite modern innovations in both diagnostic techniques as well as therapeutic regimes. Gliomas have the lowest overall survival rate compared to other cancers 5 years after definitive diagnosis. The dietary intake of vitamin C has protective effect on glioma risk. Vitamin C is an essential compound that plays a vital role in the regulation of lysyl and prolyl hydroxylase activity. Neurons store high levels of vitamin C via sodium dependent-vitamin C transporters (SVCTs) to protect them from oxidative ischemia-reperfusion injury. Vitamin C is a water-soluble enzyme, typically seen as a powerful antioxidant in plants as well as animals. The key function of vitamin C is the inhibition of redox imbalance from reactive oxygen species produced via the stimulation of glutamate receptors. Gliomas absorb vitamin C primarily via its oxidized dehydroascorbate form by means of GLUT 1, 3, and 4 and its reduced form, ascorbate, by SVCT2. Vitamin C is able to preserve prosthetic metal ions like Fe^2+^ and Cu^+^ in their reduced forms in several enzymatic reactions as well as scavenge free radicals in order to safeguard tissues from oxidative damage. Therapeutic concentrations of vitamin C are able to trigger H_2_O_2_ generation in glioma. High-dose combination of vitamin C and radiation has a much more profound cytotoxic effect on primary glioblastoma multiforme cells compared to normal astrocytes. Control trials are needed to validate the use of vitamin C and standardization of the doses of vitamin C in the treatment of patients with glioma.

## 1. Introduction

Gliomas represent solely primary brain cancers of glial cell or neuroepithelial origin [1–4]. Gliomas are categorized into lowest-grade tumors, lower-grade tumors, higher-grade malignancies, and highest-grade malignancies as stipulated by the American Association of Neurosurgeons [1–4]. World health organization further categorized astrocytoma into four grades [1–4]. Grade I comprises pilocytic astrocytoma; grade II comprises low-grade astrocytoma; grade III comprises anaplastic astrocytoma, whereas grade IV comprises glioblastoma multiforme (GBM) [1–4]. Grade I often has minimal transformation abilities into grades II–IV and mostly seen children. Nevertheless, grade II or III is mostly accompanied with malignant transformations into grade IV [1–4].

Vitamin C, also referred to as L-ascorbic acid/L-ascorbate, is an essential compound that plays a vital role in the regulation of lysyl and prolyl hydroxylase activity [5]. Vitamin C is a general term that describes its oxidized dehydroascorbate (DHA) and its reduced forms (ascorbate) [6]. Vitamin C is a water-soluble enzyme, typically seen as a powerful antioxidant in plants as well as animals [7]. Vitamin C was capable of preserving prosthetic metal ions like Fe^2+^ and Cu^+^ in their reduced forms in several enzymatic reactions as well as scavenge free radicals in order to safeguard tissues from oxidative damage [8, 9]. Also, vitamin C participates in numerous intracellular as well as extracellular biological processes to efficiently scavenge free radicals [7, 10].

Vitamin C intake as a dietary antioxidant was capable of augmenting growth restriction of cancer cells in general and glioma cells to be specific [11, 12]. Studies have shown that vitamin C was capable of inhibiting cancer via mechanism, such as the argumentation of stromal integrity of normal tissue, activating lymphocytes to a greater level of immunocompetence, stimulating “auspicious modification in the steroid environment,” blocking hyaluronidase activity in malignant cells, augmenting antiviral activity, and interfering with the metabolism of malignant cells [13–16].

Studies have demonstrated that vitamin C was selectively concentrated in tumors and may form cytotoxic quantities of hydrogen peroxide (H_2_O_2_) within the tumor as a byproduct of oxidation [13, 15, 16]. Vitamin C can act as a prodrug to deliver a substantial influx of H_2_O_2_ to tumors after intravenous (IV) administration [17, 18]. A study established that H_2_O_2_ was the key mediating factor in cytotoxicity to cancer cells through IV vitamin C [17, 19]. Vitamin C stimulated intracellular oxidation as well as energy generation resulting in total therapeutic potential. Also, vitamin C stimulated of activities like apoptosis and necrosis [17, 20].

Studies have shown that vitamin C precisely eradicated a sizable quantities of cancer cells when plasma concentrations reach 1 mM or more [21–23]. Another study revealed that vitamin C was capable of decreasing the adverse reactions triggered by chemotherapy during the treatment of cancer in patients [24, 25]. Thus, this explicit review explores the pivotal neurophysiologic and therapeutic potentials of vitamin C in glioma. The “Boolean logic” was used to search for article role of vitamin C in glioma. Most of the articles were indexed in PubMed and/or PMC with strict inclusion criteria being the neurophysiologic and therapeutic potentials of vitamin C in glioma. The search terms on PubMed and/or PMC were vitamin C and/or L-ascorbic acid and/or L-ascorbate and glioma.

### 1.1. Vitamin C Levels in the Blood, Cerebrospinal Fluid, and Brain

The brain, spinal cord, and adrenal glands had the highest vitamin C levels of all the tissues in the body as well as the highest retention capacity of vitamin C [26, 27]. Studies have shown that brain tissue concentration of vitamin C was regionally dependent. Higher concentrations were detected in anterior regions like the cerebral cortex as well as hippocampus, with gradually lower concentrations in more posterior regions like the brainstem as well as spinal cord [28, 29]. Generally, brain tissue vitamin C levels were several millimolars (mM) with the average concentration in neurons likely to be 10 mM and merely 1 mM in glia [28–30]. Under normal circumstances, turnover of vitamin C in brain is approximately 2% per hour [26, 31].

Molecules with low molecular weight as well as passable hydrophilic/hydrophobic balance are allowed to penetrate the central nervous system (CNS) [32, 33]. It was established that endothelial cells of the brain capillaries, which form the blood-brain barrier (BBB), possess selective transport systems for particular nutrients as well as endogenous biomolecules besides unspecific permeation [32]. Thus, they are conscientious for the transport of glucose, neutral, acidic, and basic amino acids like alanine and taurine, monocarboxylic acids, amines, and neuromediators like choline, vitamins, and nucleosides, as well as the peptide transport system for small neurotropic peptides [32, 34, 35].

**Figure 1:**
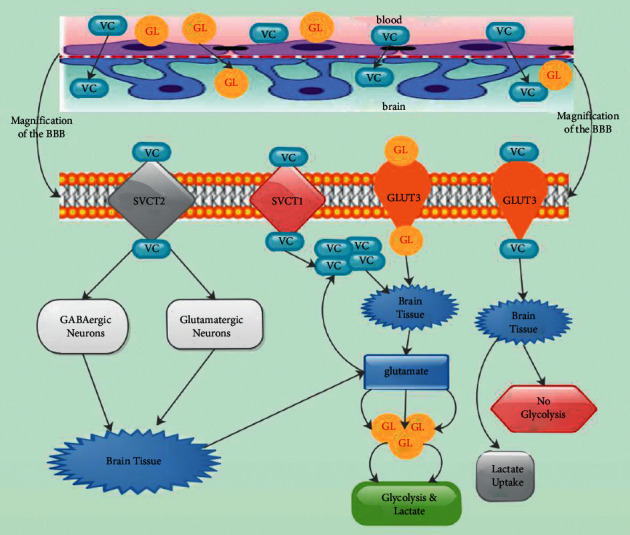
The neurophysiological mechanisms via which vitamin C and glucose cross the blood brain barrier (BBB) to influence normal brain tissues. VC = vitamin C; GL = glucose. All other abbreviations are indicated in the abbreviation list.

The epithelium of the choroid plexus, which is a restricted part of the BBB, is implicated for the maintenance of CNS homeostasis for vitamin C [32, 36]. IV administration of vitamin C revealed that vitamin C reached the CSF via the choroid plexus and then gradually penetrates the brain substance from the CSF (Figure 1) [32, 37]. Vitamin C enters the CNS principally via active transport at the choroid plexus (Figure 1). Vitamin C concentration is modulated homeostatically after it diffuses from cerebrospinal fluid (CSF) to brain extracellular fluid (ECF) [32]. Vitamin C was capable of entering the ECF via carrier-mediated uptake and via simple diffusion across brain capillaries at the BBB [26, 38]. Extracellular vitamin C levels are also vigorously regulated via glutamate-mediated activity through glutamate-vitamin C heteroexchange (Figure 1) [26].

It was established that vitamin C from ECF was taken up into brain cells, where its levels augmented up to 20-fold [26]. It was further revealed that, in some neurons, vitamin C levels were up to 200-fold higher than the levels in the bloodstream [26, 32]. Vitamin C is transferred from the blood, where its levels are about 50 *μ*M into the CFS where its levels are maintained at 200 *μ*M via specific physiological mechanisms (Figure 1) [32, 39]. Vitamin C uptake from the blood into CSF involves active stereospecific Na^+^-dependent transport at the choroid plexus (Figure 1) [26, 40]. Furthermore, vitamin C serves as a cofactor in several enzymatic activities associated with the processing of neurotransmitters as well as an antioxidant offering neuroprotection within the CNS [32].

Tsukaguchi et al. indicated that the reduced form of vitamin C is absorbed via a mechanism that involves sodium-dependent vitamin C transporters 2 (SVCT2) [8]. SVCT2 RNA was identified in the epithelium of the choroid plexus [7]. Precisely, the neuroepithelial cells of the choroid plexus as well as the retinal pigmented epithelium secreted SVCT2 transporter. It was established that SVCT1 as well as SVCT2 each mediate concentrative, high-affinity vitamin C transport that was stereospecific and was driven by the Na^+^ electrochemical gradient (Figure 1) [8]. Higher levels of Na^+^-dependent vitamin transporters such as SVCT1 and SVCT2 were detected in the choroid plexus but not in brain capillaries (Figure 1) [8, 26].

In situ hybridization in the rat brain revealed that SVCT2 was more concentrated in neurons than glial cells, which was coherent with higher concentrations of vitamin C in neurons than glia [8, 26]. Thus, neuronal cells take up vitamin C, because these cells secrete SVCT2 [41]. It was affirmed that SVCT2 was present in both glutamatergic as well as GABAergic neurons, including glutamatergic pyramidal cells of the hippocampus, glutamatergic granule cells of the cerebellum, and GABAergic cerebellar Purkinje cells (Figure 1) [8]. It was affirmed that astrocytes were capable of removing glutamate from the synaptic cleft when synapses are glutamatergically active [8].

Several studies have demonstrated that glutamate transport in these cells was capable of activating glucose transport, to stimulate glycolysis with lactate and vitamin C release (Figure 1) [42, 43]. Castro et al. demonstrated that intracellular vitamin C blocked glucose transport via direct or indirect blockade of GLUT3 and triggered lactate uptake (Figure 1) [44]. Thus, when GLUT3 was downregulated, glucose utilization was not inhibited by vitamin C and lactate transport was not stimulated (Figure 1) [42].

### 1.2. Function of Vitamin C in the Normal Brain

**Figure 2:**
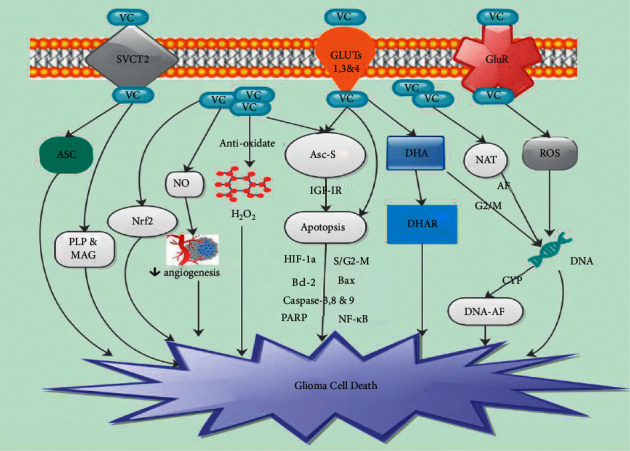
The mechanisms via which vitamin triggers glioma cell death. VC = vitamin C; ASC = ascorbate. All other abbreviations are indicated in the abbreviation list.

Brain vitamin C concentrations are gender dependent, with lower estrogen-modulated levels in the female brain than in the male brain [26, 45]. Neurons store high levels of vitamin C via SVCTs to protect them from oxidative ischemia-reperfusion injury [46, 47]. Studies have demonstrated that the key function of vitamin C was the inhibition of redox imbalance from reactive oxygen species (ROS) produced via the stimulation of glutamate receptors (Figure 2) [48, 49]. Studies have further exhibited that vitamin C was capable of buffering glutamate-generated ROS and inhibited succeeding cell death in cultured neurons [50, 51].

Studies have demonstrated that vitamin C serves as a neuromodulator for both dopamine- and glutamate-mediated neurotransmission besides its functions as an antioxidant in the CNS [52, 53]. It was further established that the main localization of vitamin C in neurons was coherent with such neuromodulatory functions [52, 53]. Furthermore, vitamin C was implicated as a fundamental cofactor for noradrenaline synthesis [26, 54]. Also, vitamin C was essential for the secretion of noradrenaline as well as acetylcholine from synaptic vesicles [26, 55]. In addition, vitamin C was a crucial cofactor in the synthesis of numerous neuropeptides [26, 56]. Moreover, at physiological concentrations, vitamin C augmented the secretion of theses neuropeptides [26, 57].

The accumulate of vitamin C in the basal lamina triggered myelin formation by Schwann cells [26, 58]. Studies have shown that variations in vitamin C concentrations were associated with brain activity as well as brain energetics [59, 60]. Also, vitamin C servers as a metabolic switch in brain, modulating glucose consumption in neuronal cells via the blockade of neuronal GLUT3 [42]. Owens and Bunge established that vitamin C was a fundamental in the enhancement of axonal ensheathment in Schwann cell-neuronal coculture [61]. They further revealed that vitamin C was crucial for periphery nervous system myelinogenesis because it was capable of stimulating the P0 protein gene in cultured Schwann cells [61].

### 1.3. Vitamin C and Glioma

Several studies have assessed the outcomes of dietary vitamin C intake on primary brain tumor risk [62–70]. These studies involved both children and adults [62–70]. The studies in children revealed that glioma risk differs inversely with maternal vitamin C intake during pregnancy [62–64]. In studies involving adults, an inverse association between dietary vitamin C intake and glioma risk was also observed [62, 65–67]. Nevertheless, some studies detected a positive relationship between vitamin C intake and glioma risk [62, 68, 69]. Another study found positive relationship for men and a negative relationship for women [70]. Zhou et al. with a meta-analysis observed that the consumption of vitamin C had protective effect on glioma risk [11].

Studies have shown that GLUTs mediate the facilitative transport of the DHA form of vitamin C [6, 71, 72]. Also, studies have demonstrated that ascorbate, the reduced form of vitamin C, is transported by SVCT [6, 8, 73, 74]. Gliomas absorb vitamin C primarily via its oxidized form (DHA) by means of GLUT 1, 3, and 4 and its reduced form, ascorbate, by SVCT2 (Figure 2) [6]. Nevertheless, it was established that SVCT2 had modest capacity in gliomas [75]. DHA was reduced to vitamin C via the GSH-consumption enzyme DHA reductase (DHAR) once it gets into the cells (Figure 2) [6]. Laszkiewicz et al. exhibited that, vitamin C is a potent modulator of the proteolipid protein as well as the secretion of myelin-associated glycoprotein gene in CNS-derived C6 cells [76].

Salmaso et al. established that vitamin C could be utilized as a targeting agent to stimulate the disposition of drug loaded nanosystems in gliomas [32]. Conklin et al. indicated that antioxidants are capable of safeguarding normal brain tissues from radiation damage resulting in better survival, because brain tissues possess oxidative milieus and are thus susceptible to radiation damage [77]. Lawenda et al. demonstrated that antioxidants are capable of rending glioma more resistant to tumor killing by radiation, resulting in poorer patient survival [78]. It was established that, at low doses, vitamin C was capable of protecting cells from oxidative stress, thus inhibiting the advancement of tumors [5, 79]. Prasad demonstrated that sodium ascorbate triggered a cytotoxic stimulus on normal brain cells in culture [15]. Benade et al. established that the toxicity of ascorbate was as a result of low catalase levels in tumor cells [80].

Vitamin C was able to inhibit DNA damage and the deterioration of subcellular structures like proteins, lipids, and DNA by scavenging of ROS (Figure 2) [5]. Studies have demonstrated that therapeutic concentrations of vitamin C triggered H_2_O_2_ generation in solid tumors (Figure 2) [22, 81]. Furthermore, studies have demonstrated that H_2_O_2_ diffuses into cancer cells and overpowers their antioxidant defense system via the depletion of glutathione levels [22, 23]. Espey et al. established that vitamin C stimulated generation of extracellular H_2_O_2_ was only partly accountable for cell death [82]. Peterkofsky and Prather indicated that H_2_O_2_ was either formed intracellularly and excreted in the medium or formed at the cell surface on culture medium [83].

It was further established that H_2_O_2_ was not detectable in growth medium containing Na^+^-ascorbate alone [83]. Studies have demonstrated that H_2_O_2_ was capable of triggering lipid peroxidation, which resulted in cell death [84–86]. It is also revealed that Na^+^-ascorbate was capable of triggering the formation of DHA exogenously or intracellularly or both. Also, sodium ascorbate was capable of blocking catalase activity *in vitro* [87]. Furthermore, the blockade of catalase was capable stimulating the accumulation of H_2_O_2_ in tumor cells resulting in cell death [87].

### 1.4. Vitamin C Derivatives and Glioma

Ascorbyl esters are nontoxic, synthetic derivative of vitamin C used as antioxidants [88, 89].

These esters can easily cross the BBB because of their lipophilic nature [90]. The breakdown products of ascorbyl stearate are ascorbate and stearic acids, which are nontoxic to biological system [88, 89]. Studies have indicated that ascorbyl esters, such as ascorbyl stearate (Asc-S) and ascorbyl palmitate, block the proliferation of mouse as well as human glioma cells [91, 92]. Furthermore, studies have demonstrated that Asc-S as well as ascorbyl palmitate suppressed the growth of murine (G-26) as well as human glioma (U-373) cells [91, 92]. Makino et al. established that Asc-S, a lipophilic derivative of vitamin C is a potent inhibitor of cell proliferation as compared to vitamin C [93].

Studies have demonstrated that human gliomas are capable of secreting insulin-like growth factor- (IGF-) I as well as IGF-II. It was further established that IGFs autocrine receptor was capable of stimulating glioma cell growth [94, 95]. Naidu et al. established that Asc-S was capable of modulating of secretion of IGF-IR as well as triggering of apoptosis in T98G cells (Figure 2) [96]. They revealed that Asc-S inhibited the growth of human GBM T98G cells via the arrest of cells at late S/G2-M phase of cell cycle as well as trigger cell death via apoptosis (Figure 2) [96]. They further indicated that Na^+^-ascorbate was capable of blocking the growth of T98G cells with an IC50 of 6.0 mM [96]. Nevertheless, Asc-S was about 68-fold more potent than Na^+^-ascorbate with an IC50 value of 88.5 *μ*M [93].

It was also established that administration of Asc-S led to a substantial augmentation in the proportion of cells in late S/G2-M phase of cell cycle in comparison with untreated control cells [96]. Also, DHA was capable of modulating cell cycle progression as well as trigger cell cycle arrest at G2/M DNA damage checkpoints during oxidative stress (Figure 2) [97]. Furthermore, Asc-S stimulated cell cycle arrest at late S/G2-M phase checkpoints was capable of blocking of cell proliferation as well as apoptosis [96]. Thus, vitamin C derivatives interfere with cell cycle progression [96]. Ryszawy et al. demonstrated that Na^+^-ascorbate was capable of triggering significant impairment of GBM cell viability as well as invasiveness [5].

Also, the blockade influence of Na^+^-ascorbate on GBM cell motility resulted in heterogeneous viability-associated cell responses [5]. Moreover, a rapid necrotic-like death was detected in a proportion of cells with Na^+^-ascorbate, which resulted in cell swelling, membrane break, and their release from cytoplasm [5]. Furthermore, “autoschizis”-associated violent cell responses to elevated Na^+^-ascorbate doses substituted apoptosis in “hypersensitive” GBM cells [5]. This cell death mechanism was a self-excision of cytoplasm and was detected only in the coexistence of vitamin C and menadione [98, 99].

### 1.5. Vitamin C and Glioma Angiogenesis

Angiogenesis is a normal physiological activity, obligatory for normal tissue repair as well as growth [100]. Nevertheless, angiogenesis is depicted by the assiduous proliferation of endothelial cells as well as blood vessel formation in pathological situations [100]. Thus, angiogenesis is very critical in tumor growth, invasion, and metastasis [100]. Studies have implicated the association of circulating endothelial precursor cells (EPCs) to pathologic angiogenesis [101–103]. Several studies have demonstrated that nitric oxide (NO) was associated with tumor angiogenesis [104–106].

Dulak et al. demonstrated that NO was capable of modulating for the secretion of endogenous angiogenic factors like vascular endothelial growth factor (VEGF) as well as basic fibroblast growth factor (bFGF) [107]. Studies established that tumors that produced NO persistently had significantly supplementary vascular network and were more invasive [108, 109]. Thus, angiogenesis is determined by the level of NO, which also influence migration as well as precise motivity of the endothelial cells [100, 110].

Telang et al. analyzed the effect of vitamin C on tumor development in animals after dietary consumption of low levels [111]. Peyman et al. established that the total number of blood vessels were decreased in vitamin C depleted tumors compared to the totally supplemented animals. Contrariwise, high levels of vitamin C administered to cauterized corneas suppressed angiogenesis in a rat prototype [112]. Mikirova et al. evaluated the effect of high levels of vitamin C (100 mg/dl–300 mg/dl) on *in vitro* endothelial cells as well as new blood vessel formation [100]. They observed that IV administration of 25–60 grams of vitamin C affect both endothelial progenitor cells as well as mature endothelial cell functions associated with process of angiogenesis (Figure 2) [100].

Furthermore, the effect of vitamin C on angiogenesis assessed via tube formation assay exhibited blockade of vessel structure after 3–24 h of exposure of the cells to vitamin C (Figure 2) [100]. This appeared as a result of vitamin C ability to block NO in endothelial cells (Figure 2) [100]. Duda et al. established that NO is a key stimulus of new blood vessel formation [113]. Thus, vitamin C was capable of inhibiting NO stimulation resulting in the inhibition of angiogenesis as well as vasculogenesis (Figure 2) [113].

### 1.6. Signaling Pathways of Vitamin C in Glioma

Vitamin C was implicated in several signal pathways associated with the development of glioma [114–119]. Vitamin C had much a stronger influence on the crucial stages of tumor cell proliferation as well as differentiation by shifting their epigenome and transcriptome. Naidu et al. observed antiproliferative as well as apoptotic effects of vitamin C on T98G glioma cells via modulation of IGF-IR secretion subsequent to the facilitation of programmed cell death [96]. Also, vitamin C was capable of upregulating proteolipid protein (PLP) as well as myelin-associated glycoprotein (MAG) genes in glioma C6 cells of rat models (Figure 2) [76].

Nuclear factor erythroid 2-related factor 2 (Nrf2) is a fundamental constituent of cellular defense against a wide range of endogenous as well as exogenous stresses [114]. It was observed that vitamin C was capable of influencing Nrf2 in GBM (Figure 2) [114]. Hypoxia-inducible factor 1*α* (HIF-1*α*) is a transcription factor responsible for the cellular reaction to low O_2_ conditions via the modulation of genes regulating various cellular transduction pathways [115]. HIF-1*α* further modulates growth and apoptosis, cell migration, energy metabolism, angiogenesis, and transport of metal ions and glucose [115]. HIF-1*α* is often intensely oversecreted in common cancers, cancer cell lines, and metastases [116].

Several studies have demonstrated that therapeutic levels of vitamin C downregulated cell survival pathways in cancer cells via HIF-1*α* as well as the nuclear transcription factor (NF-*κ*B) [117–119]. Vitamin C was capable of regulating HIF-1*α* in common cancers including glioma [120]. Also, vitamin C was able to promote prolyl as well as lysyl hydroxylases in the hydroxylation of HIF-1*α* (Figure 2) [120]. It was established that low vitamin C levels were able to decrease HIF-1*α* hydroxylation resulting in the promotion of HIF-dependent gene transcription as well as tumor growth [120]. Bi et al. established that over secretion of Bcl-2 and blockade of Bax secretion correlated well with antiapoptosis/apoptosis imbalance of glioma cells (Figure 2) [121].

Duan et al. demonstrated that Maitake mushroom (MP)/vitamin C was able to inhibit the proliferation of glioma cells, augmented tumor cell apoptosis, and reduced mRNA/protein secretion of Bcl-2 while augmenting Bax mRNA or protein secretion (Figure 2) [7]. They further observed augmentation in the secretion of caspase-3 as well as its endogenous substrate, cleavage of PARP [7]. Moreover, MP/vitamin C was able activate key mediators of the apoptosis pathway, such as caspase-3, caspase-8, and caspase-9 in M059 K cells (Figure 2) [7]. Thus, the combination of MP and VC triggered M059 K cell apoptosis [7]. Holme et al. revealed that vitamin C was capable of decreasing the cytotoxic properties of N-hydroxy-acetylaminofluorene and decrease the covalent binding of N-acetyl-2-aminofluorene (AAF) to cellular protein [122]. Further studies are needed to establish the effect of vitamin C on this protein in glioma.

Hung established that rat glial tumor cells possess N-acetyltransferase (NAT) properties [123]. Furthermore, the rat's brain tissue was able to modulate NAT activity as well as the stimulation of N-acetylation of 2-aminofluorene (AF) (Figure 2) [124]. Hung and Lu demonstrated that vitamin C was able to block NAT activity in C6 glioma cells [125]. They also revealed that vitamin C reduced AF-DNA adduct formation in C6 glioma cells, but vitamin C did not influence DNA to transcript NAT mRNA [125]. Miller and Miller showed that AF is N-acetylated via NAT and subsequently metabolized via cytochrome P450 (CYP) into a reactive metabolite, which binds to DNA to form DNA-AF metabolite adduct (Figure 2) [126].

### 1.7. Vitamin C in Glioma Treatment

Cameron and Pauling in 1976 suggested that IV vitamin C followed by oral maintenance was a beneficial therapy for patients with cancer [127]. Thus, vitamin C, specifically at high therapeutic levels, has a long and widely been used as cancer treatment in history [127, 128]. IV vitamin C was demonstrated to be toxic to tumor cells, but not to normal cells [129]. Furthermore, IV vitamin C was capable of inhibiting angiogenesis and inflammation, boosts the immune system, causes differentiation of cells, and improves quality of life of patients with cancer [100].

Currently, temozolomide is the drug of choice for the management of patients with glioma [24, 25, 130]. It is an orally bioavailable, methylating agent that is able to pass through the BBB and trigger the death of tumor cells [24, 25]. Nevertheless, some tumor cells are capable of repairing DNA damage triggered by temozolomide and thus lessen the efficiency of the therapy [24, 25]. Laboratory and clinical studies have demonstrated that temozolomide's anticancer efficiency was augmented when combined with etoposide [24, 25].

Gokturk et al. demonstrated that vitamin C alone was capable of triggering oxidative DNA damage in glioma [130]. They revealed that cytotoxic as well as genotoxic effects of temozolomide and etoposide were reduced by vitamin C, but the utmost cytotoxicity with the least genotoxicity was attained with use of the triple therapy [130]. Thus, vitamin C reduced the cytotoxic as well as genotoxic effect of the etoposide and etoposide-temozolomide combination, but it had no significant effect on temozolomide's toxicity [130].

Mikirova et al. were able to treat neurofibromatosis type 1 (NF1) patient with optic pathway glioma (OPG) with a high dose of IV vitamin C [131]. They suggested that vitamin C treatment may be appropriate for young patients' glioma who are not suitable to receive standard treatments regimes due to their toxicity [131]. Studies have demonstrated that radiotherapy offers a 6-month survival benefit at a median time frame for glioma patients [132, 133]. Herst et al. demonstrated that radiation dose of 2-Gy fractions alone for GBM patients and vitamin C alone at concentrations >1 mM was effective for GBM patients [21].

Herst et al. indicated further that combination therapy using 0.5 mM vitamin C and lower radiation dose of 1-Gy fraction killed considerably more primary GBM cells and astrocytoma cells compared with single therapy [21]. Nevertheless, the combination therapy had a much lesser effect on normal astrocytes, suggesting a certain level of specificity for GBM cells [21]. Thus, they study exhibited that in the clinical situation, combination therapy triggers more specific GBM killing with lower doses of radiation as well as less damage to adjacent, healthy tissues [21].

Herst et al. demonstrated that administration of vitamin C was capable of inhibiting radiation-stimulated G2/M arrest in GBM primary cells, but not in astrocytes, inhibiting homologous recombination and hence DSB repair, which was specifically poor in GBM cells compared with normal astrocytes [21]. Furthermore, both vitamin C and radiation therapy were able to trigger cell death associated with autophagy [134]. Autophagy is a salvaging mechanism that is stimulated in cells under stress [135]. Studies have demonstrated that 5 mM vitamin C, 6-Gy fractions, or combined therapy did not trigger apoptotic cell death in GBM primary cell [134, 136].

Herst et al. postulated that our cells primarily use autophagy as a survival mechanism after exposure to radiation, vitamin C, or combined treatment [21]. They concluded that high-dose combination of vitamin C and radiation has a much more profound cytotoxic effect on primary GBM cells compared to normal astrocytes, and this combination could be a safe as well as clinically viable alternative for treating aggressive radiation-resistant GBMs [21]. Prasad et al. report that vitamin C at nontoxic doses potentiated growth inhibitory capabilities of 5-fluorouracil (5-FUra), bleomycin sulfate, sodium butyrate, cyclic AMP stimulating agents, and X-irradiation on neuroblastoma (NB) cells, but it did not yield analogous capabilities on rat glioma cells in culture [137].

Prasad et al. further postulated that if vitamin C is used arbitrarily in combined therapies, it may reduce the efficiency of some chemotherapeutic agents [137]. They indicated that vitamin C was capable of reducing the cytotoxic effect of methotrexate as well as 5-(3,3-dimethyl-btriazeno)-imidazole-4-carboxamide (DTIC) on NB cells in culture [137]. This was perhaps due to deactivation of these medicines *in vitro* by vitamin C [137]. Prasad et al. in another study demonstrated that vitamin C at nontoxic doses significantly potentiated the effect of methylmercuric chloride (MMC) on NB cells while it did not alter the effect of MMC on glioma cells [137].

The effect of vitamin C was most distinct at a MMC doses of 1 *μ*M [137]. Moreover, vitamin C was similarly effective in potentiating the effect of MMC on NB cells, but glutathione did not exhibit similar effect [137]. Schoenfeld et al. demonstrate that increased labile Fe^2+^ pool levels, triggered by mitochondrial superoxide and H_2_O_2_, expressively participated in cancer cell-selective toxicity of therapeutic vitamin C combined with standard radio-chemotherapy in GBM models [138]. They postulated that augmented labile Fe^2+^ in cancer cells triggered an upsurge in oxidation of vitamin C to produce H_2_O_2_ capable of further aggravating the differences in labile Fe^2+^ in cancer compared to normal cells [138].

The above occurred, at least partly, because of H_2_O_2_-mediated interference of Fe-S cluster-containing proteins [138]. The augmented levels of H_2_O_2_, in the company of an augmented labile Fe^2+^ pool, triggered an upsurge in Fenton chemistry to produce hydroxyl radicals resulting in oxidative damage [138]. Sharma and Khanna showed that vitamin C inhibited etorphine-stimulated compensatory upsurge in the concentrations of cyclic AMP with slight or no influence on the temporary response of NG108-15 hybrid cells to the effector agents, but it had no effect on the temporary blockade response of the cells to the drug [139].

Sharma and Khanna suggest the potential use of vitamin C in the prevention of the development of tolerance in therapeutic usage of narcotics as analgesics [139]. Vita et al. demonstrated that menadione alone or in combination with vitamin C exhibited similar concentration-response curves as well as IC50 values [140]. They indicated that menadione: vitamin C at a ratio of 1 : 100 exhibited higher antiproliferative activity when compared to each medicine alone and permitted to decrease each medicine concentration between 2.5 and 5-fold [140]. Analogous antiproliferative effects were exhibited in 8 patients derived GBM cell cultures [140].

## 2. Conclusion

The dietary intake of vitamin C has protective effect on glioma risk. Neurons store high levels of vitamin C via SVCTs to protect them from oxidative ischemia-reperfusion injury. The key function of vitamin C is the inhibition of redox imbalance from ROS produced via the stimulation of glutamate receptors. Vitamin C is able to inhibit DNA damage and the deterioration of subcellular structures like proteins, lipids, and DNA by scavenging of ROS. Also, therapeutic concentrations of vitamin C are capable of triggering H_2_O_2_ generation in solid tumors including glioma. The total number of blood vessels was decreased in vitamin C depleted tumors compared to the totally supplemented animals, which means that vitamin C is capable of inhibiting tumor angiogenesis. High-dose combination of vitamin C and radiation has a much more profound cytotoxic effect on primary GBM cells compared to normal astrocytes, and this combination could be a safe as well as clinically viable alternative for treating aggressive radiation-resistant GBMs. Control trials are needed to validate the use of vitamin C and standardization of the doses of vitamin C in the treatment of patients with glioma.

## Data Availability

No data were used in this paper.

## Abbreviations

AF: 2-Aminofluorene
5-FUra: 5-Fluorouracil
DTIC: 5-(3,3-Dimethyl-btriazeno)-imidazole-4-carboxamide
Asc-S: Ascorbyl stearate
BBB: Blood-brain barrier
bFGF: Basic fibroblast growth factor
CNS: Central nervous system
CSF: Cerebrospinal fluid
CYP: Cytochrome P450
DHA: Dehydroascorbate
DHAR: DHA reductase
DNA: Deoxyribonucleic acid
ECF: Extracellular fluid
EPCs: Endothelial precursor cells
GBM: Glioblastoma multiforme
H_2_O_2_: Hydrogen peroxide
HIF-1*α*: Hypoxia-inducible factor 1*α*
IV: Intravenous
IGF: Insulin-like growth factor
mM: Millimolar
MAG: Myelin-associated glycoprotein
MMC: Methylmercuric chloride
NO: Nitric oxide
Nrf2: Nuclear factor erythroid 2-related factor 2
NF-*κ*B: Nuclear transcription factor
NAT: N-acetyltransferase
NF1: Neurofibromatosis type 1
NB: Neuroblastoma
OPG: Optic pathway glioma
PLP: Proteolipid protein
ROS: Reactive oxygen species
SVCTs: Sodium-dependent vitamin C transporters
VEGF: Vascular endothelial growth factor.
